# TRIOBP-1 Protein Aggregation Exists in Both Major Depressive Disorder and Schizophrenia, and Can Occur through Two Distinct Regions of the Protein

**DOI:** 10.3390/ijms231911048

**Published:** 2022-09-21

**Authors:** Beti Zaharija, Maja Odorčić, Anja Hart, Bobana Samardžija, Rita Marreiros, Ingrid Prikulis, Maja Juković, Thomas M. Hyde, Joel E. Kleinman, Carsten Korth, Nicholas J. Bradshaw

**Affiliations:** 1 Department of Biotechnology, University of Rijeka, 51000 Rijeka, Croatia; 2 Department of Neuropathology, Heinrich Heine University, 40225 Düsseldorf, Germany; 3 Lieber Institute for Brain Development, Baltimore, MD 21295, USA; 4 Department of Psychiatry and Behavioral Sciences, John Hopkins University School of Medicine, Baltimore, MD 21205, USA; 5 Department of Neurology, John Hopkins University School of Medicine, Baltimore, MD 21287, USA

**Keywords:** domain structure, major depressive disorder, protein aggregation, proteinopathy, schizophrenia, TRIOBP-1

## Abstract

The presence of proteinopathy, the accumulation of specific proteins as aggregates in neurons, is an emerging aspect of the pathology of schizophrenia and other major mental illnesses. Among the initial proteins implicated in forming such aggregates in these conditions is Trio and F-actin Binding Protein isoform 1 (TRIOBP-1), a ubiquitously expressed protein involved in the stabilization of the actin cytoskeleton. Here we investigate the insolubility of TRIOBP-1, as an indicator of aggregation, in brain samples from 25 schizophrenia patients, 25 major depressive disorder patients and 50 control individuals (anterior cingulate cortex, BA23). Strikingly, insoluble TRIOBP-1 is considerably more prevalent in both of these conditions than in controls, further implicating TRIOBP-1 aggregation in schizophrenia and indicating a role in major depressive disorder. These results were only seen using a high stringency insolubility assay (previously used to study DISC1 and other proteins), but not a lower stringency assay that would be expected to also detect functional, actin-bound TRIOBP-1. Previously, we have also determined that a region of 25 amino acids in the center of this protein is critical for its ability to form aggregates. Here we attempt to refine this further, through the expression of various truncated mutant TRIOBP-1 vectors in neuroblastoma cells and examining their aggregation. In this way, it was possible to narrow down the aggregation-critical region of TRIOBP-1 to just 8 amino acids (333–340 of the 652 amino acid-long TRIOBP-1). Surprisingly our results suggested that a second section of TRIOBP-1 is also capable of independently inducing aggregation: the optionally expressed 59 amino acids at the extreme N-terminus of the protein. As a result, the 597 amino acid long version of TRIOBP-1 (also referred to as “Tara” or “TAP68”) has reduced potential to form aggregates. The presence of insoluble TRIOBP-1 in brain samples from patients, combined with insight into the mechanism of aggregation of TRIOBP-1 and generation of an aggregation-resistant mutant TRIOBP-1 that lacks both these regions, will be of significant use in further investigating the mechanism and consequences of TRIOBP-1 aggregation in major mental illness.

## 1. Introduction

Schizophrenia, like many chronic mental illnesses, has a highly complex pathophysiology, epitomized by the large number of genetic variants that contribute to it [1], making it difficult to identify specific protein targets for diagnosis or therapy. Therefore, in a complementary approach, we and others have been investigating proteinopathy as a possible physiological aspect of this condition [2,3]. This is based on the hypothesis that specific proteins may become aggregated in neurons of schizophrenia patients and impede their function, in partial analogy to neurotoxic aggregates of specific proteins in the neurodegenerative disorders [3]. Notably, it has now been demonstrated that subgroups of schizophrenia patients display impaired proteasomal activity, leading to the accumulation of misfolded, insoluble protein [4,5,6]. Several proteins have now been identified that potentially aggregate specifically in schizophrenia or major mental illness more generally [2,7,8,9,10].

Among the proteins implicated as aggregating in schizophrenia is Trio and F-actin Binding Protein (TRIOBP) [9]. The *TRIOBP* gene is complex and encodes multiple differentially spliced transcripts, not all of which overlap with each other [11,12,13,14]. Of these, the TRIOBP-1 isoform, encoded at the 3′ end of the *TRIOBP* gene, was identified as seemingly being specifically insoluble in brain samples of schizophrenia patients as opposed to controls, using an antibody-based proteomics approach [8,9]. TRIOBP-1 was later shown to form insoluble aggregates in a variety of systems [9,15,16,17]. Functionally, TRIOBP-1 is a ubiquitously expressed key modulator of actin [11], which binds to F-actin and prevents its depolymerization [15]. It has roles in cellular migration [18], neurite outgrowth [19], stress fiber formation in the heart [20] and interactions between the cytoskeleton, cell membrane and extracellular matrix [21,22]. It is also possible that the product of some long splice variants of *TRIOBP* can also form aggregates, but this has not been extensively investigated [9,14,17].

**Figure 1:**
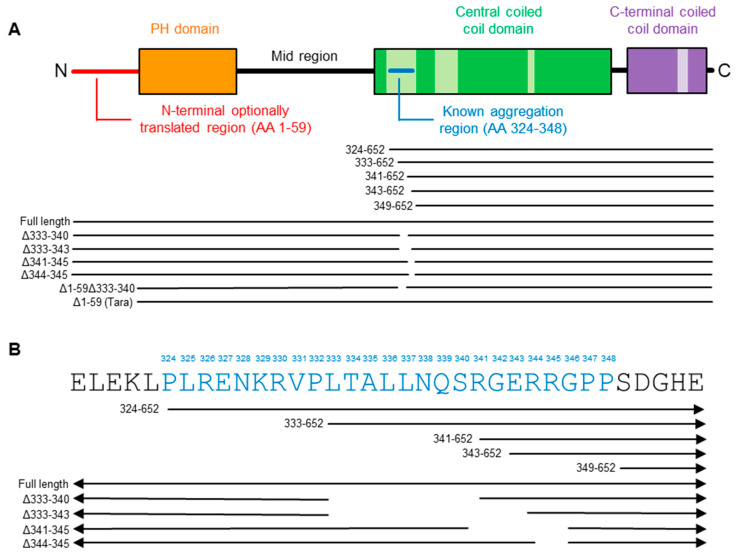
The known domain structure and aggregation critical region of TRIOBP-1 (Trio and F-actin Binding Protein isoform 1) (**A**) The domain structure of TRIOBP-1, shown in a one-dimensional format, as determined previously [11,14,15,23]. In coiled-coil domains, predicted coils are shown in darker shades, while loops are shown in paler shades. Amino acid (AA) numbers refer to the 652 AA form of TRIOBP-1, which includes the optionally translated region. (**B**) A section of the primary structure of TRIOBP-1, with the amino acids previously implicated in protein aggregation [15] shown in blue. Below each image are shown the regions of this protein contained in each of the major TRIOBP-1 plasmids used in this paper. More details of these plasmids can be found in Supplementary Table S1.

The TRIOBP-1 protein consists of three folded domains: A Pleckstrin homology (PH) domain near its N-terminus [11,14] and two coiled-coil domains in its C-terminal half [11,15,23] (Figure 1A). Of these, the larger central coiled-coil (CCC) domain is responsible for oligomerization [15]. The “mid” region between the PH and CCC domains is believed to be unstructured. Additionally, TRIOBP-1 possess two potential Kozak sequences and start codons, meaning that some TRIOBP-1 species contain an additional 59-amino acid long optionally translated region at their N-terminus, which is also predicted to be unstructured [15,24]. The shorter version of TRIOBP-1, lacking this region and thus being 593 amino acids long, was the first TRIOBP variant to be identified, and is also referred to as “Tara” or “TAP68” [11,23]. In this paper, the amino acid numbering of TRIOBP-1 will be made from the first methionine, meaning that the two TRIOBP-1 variants consist of amino acids 1-652 and 60-652 respectively.

Initial bioinformatic analyses of TRIOBP-1 suggested that its aggregation was likely driven by its PH domain, which contains several stretches of amino acids that may make it prone to aggregation. However, deletion of the PH domain from plasmid constructs encoding full-length mouse Triobp-1 did not prevent the proteins they encoded from aggregating [9]. Instead, studies using human TRIOBP-1 constructs revealed that a site within the CCC domain was likely to be responsible [15]. Specifically, the expression of amino acids (AA) 324-652 of TRIOBP-1, including all the structured C-terminal half of the protein except for one coiled-coil of the CCC domain, formed aggregates resembling the full-length protein. In contrast, a construct encoding AA 349-652, containing all of the same coiled-coils but lacking one unstructured loop region, did not form visible aggregates and distributed in the cytoplasm [15]. In support of this, while a construct consisting of AA 190-652 of TRIOBP-1 (the entire protein excepting the optionally translated region and PH domain) formed aggregates, an equivalent construct from which amino acids 324-348 (Figure 1B) had been deleted did not [15]. It was therefore concluded that this 25 amino acid-long loop, or a subpart of it, was responsible for the aggregation of TRIOBP-1.

In this work, we sought to further refine the aggregation critical region of TRIOBP-1 and found, surprisingly, that two separate parts of TRIOBP-1 are capable of inducing aggregation: a refined version of this central aggregation motif, but also the N-terminal untranslated region.

## 2. Results

### 2.1. Insoluble TRIOBP-1 Is Found in the Brains of Patients with Major Depressive Disorder and Schizophrenia, Indicating Its Aggregation in Both Conditions

In order to confirm whether aggregated TRIOBP is present in the brains of patients with major mental illness, we optimized two previously published protocols [2,25] for isolating the insoluble protein fraction of brain tissue (Supplementary Figures S1 and S2) which will be referred to as the high and low stringency purification techniques. The low stringency protocol, which was developed to enhance sensitivity, purifies both proteins known to form unfolded aggregates (as confirmed by looking at DISC1 and Tau in the brains of established transgenic rodent lines [26,27]) as well as actin (Supplementary Figure S2). Notably, actin is known to be found in the insoluble protein fraction of various assays as a result of its existence as highly multimeric fibrous F-actin, thus being an example of a protein that can be characterized as being “insoluble” despite existing in a correctly folded state.

**Figure 2:**
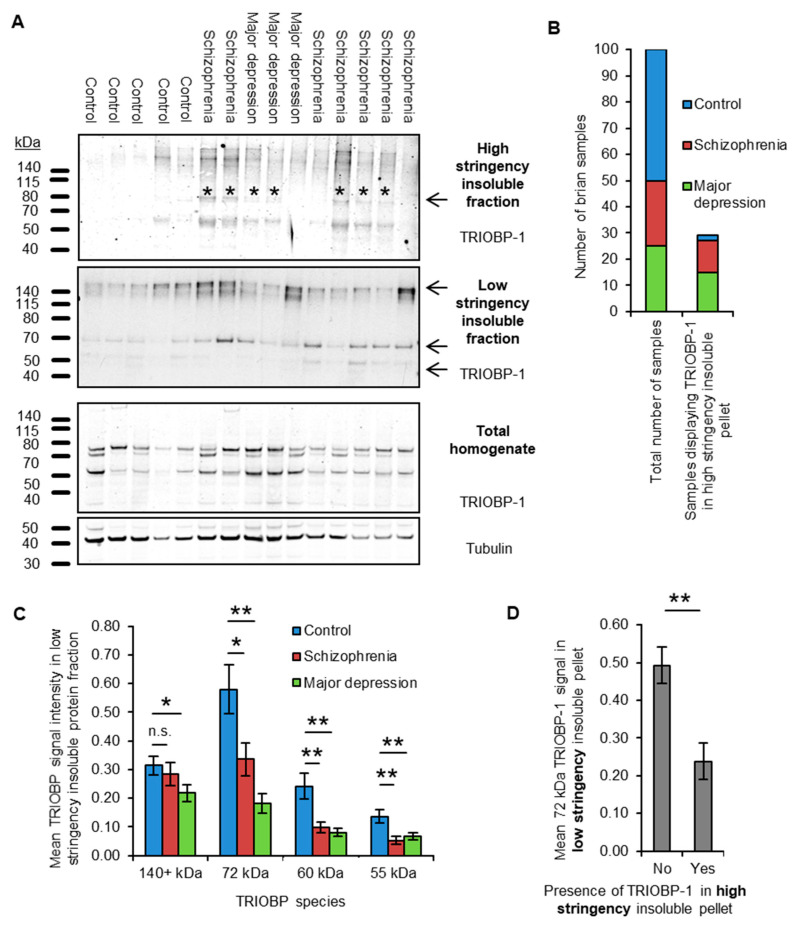
(**A**) Sample Western blot insoluble protein fractions of brain samples, derived using the high and low stringency purification protocols. Total homogenate is also shown. 15 of 100 samples in total are displayed. The protein species taken forward for further analysis are indicated by arrows. For the aggregated protein fraction, samples which were rated (in blinded analysis) as containing a TRIOBP-1 72 kDa species are indicated by an asterisk. Note that, for the sake of maximum clarity, different antibody concentrations and exposure settings were used for the blot of each fraction. (**B**) Bar graph showing the distribution of control, schizophrenia and major depression in both the complete set of 100 samples, and in the 29 which were rated as having TRIOBP-1 (72 kDa signal) in the high stringency insoluble fraction, demonstrating a significant enrichment in both mental illness groups. (**C**) Quantification of the mean signal strength in the low stringency insoluble protein fraction, for each of the four examined TRIOBP-1 species. Signal strength was normalized to the mean total TRIOBP-1 signal across all samples. (**D**) Comparison of the total level of 72 kDa TRIOBP-1 in low stringency insoluble protein fractions, between brain samples that either did or did not contain 72 kDa TRIOBP-1 in their high stringency insoluble fraction. * *p* < 0.05, ** *p* < 0.01, n.s.: not significant.

In contrast, the high stringency protocol, which was developed for enhanced specificity, seemingly detects only unfolded insoluble protein, detecting aggregated DISC1 and to an extent aggregated Tau, but not actin (Supplementary Figure S2). These protocols were used to purify the high and low stringency insoluble pellets of post mortem brain samples (anterior cingulate cortex, BA23) from 25 schizophrenia patients, 25 patients with major depressive disorder and from 50 control individuals (see Supplementary Table S1 for demographic information). Western blotting of the ensuing fractions, in a blinded manner, was then used to determine the presence of insoluble TRIOBP in these samples (Figure 2A).

The high stringency insoluble protein fraction revealed TRIOBP signal in a significant proportion of samples; however, the strength of the signal was too low to accurately quantify (Figure 2A). Each sample was therefore rated as either containing or not containing a distinguishable 72 kDa TRIOBP band, corresponding to full length TRIOBP-1 which is implicated as aggregating in schizophrenia [9]. This band was present in the high stringency insoluble fraction of 12/25 schizophrenia samples and 15/25 major depression samples (48% and 60% respectively), but only 2/50 control samples (4%, Figure 2B), implying a substantial enrichment of aggregated TRIOBP-1 in the brains of patients with either of these conditions.

In the low stringency insoluble protein pellets, four major TRIOBP bands could be easily detected (Figure 2A). The longest of these was over 140 kDa in size and is consistent with the long TRIOBP-5 or 6 splice variants, which incorporate almost the entire length of TRIOBP-1 [12,13,14]. The 72 kDa full length TRIOBP-1 band was also seen in addition to two smaller species of 60 kDa and 55 kDa, which would either correspond to breakdown products or shorter splice variants of TRIOBP. Levels of the TRIOBP-1 species in the low stringency insoluble fraction were decreased (by approximately 50%) in the brain samples from both schizophrenia and major depression patients, when compared to the control group (Figure 2C). The TRIOBP-5/6 band did not consistently vary with diagnosis. There was no correlation between TRIOBP-1 signal and post mortem interval or age of the patient at death, and only a weak correlation with the pH of the brain sample (Pearson correlation coefficient 0.249, *p* = 0.012, Supplementary Figure S3A). Ethnicity may also have an effect; however, low sample numbers make it difficult to be certain (Supplementary Figure S3B).

The result that low stringency-derived insoluble TRIOBP-1 is decreased in mental illness contrasts with the increase in high stringency-derived aggregated TRIOBP-1 in the same samples. Indeed, brain samples displaying TRIOBP-1 in their high stringency insoluble pellet, regardless of diagnosis, showed on average less than 50% of the amount of the same protein band in their low stringency insoluble fraction, with an inverse correlation existing between the two (point-biserial correlation coefficient: −0.349, *p* = 3.7 × 10^4^, Figure 2D). Therefore, while TRIOBP generally exists assembled into a high molecular weight complex, presumably associated with F-actin as part of its physiological role in actin modulation [11,14,15], it appears that in some individuals with mental illness, a fraction of this TRIOBP-1 is converted to an unfolded aggregated state (detectable using the high stringency protocol).

There was no association between the level of any TRIOBP species detectable in the total brain homogenate with mental illness, nor between levels of total and insoluble TRIOBP, as detected using either protocol (Supplementary Figure S4A–D). Notably, however, the main 72 kDa form of TRIOBP-1 was seen as a doublet in the brain homogenate, despite only one such band being seen in the insoluble fractions. The ratio between the level of expression of these two species was significantly associated with major depression (Supplementary Figure S4E). A potential explanation for this is that it represents TRIOBP-1 protein that either has or is lacking the 59 AA optional N-terminal domain.

### 2.2. Refining the Aggregation Domain of TRIOBP-1, Using C-Terminal Constructs

**Figure 3:**
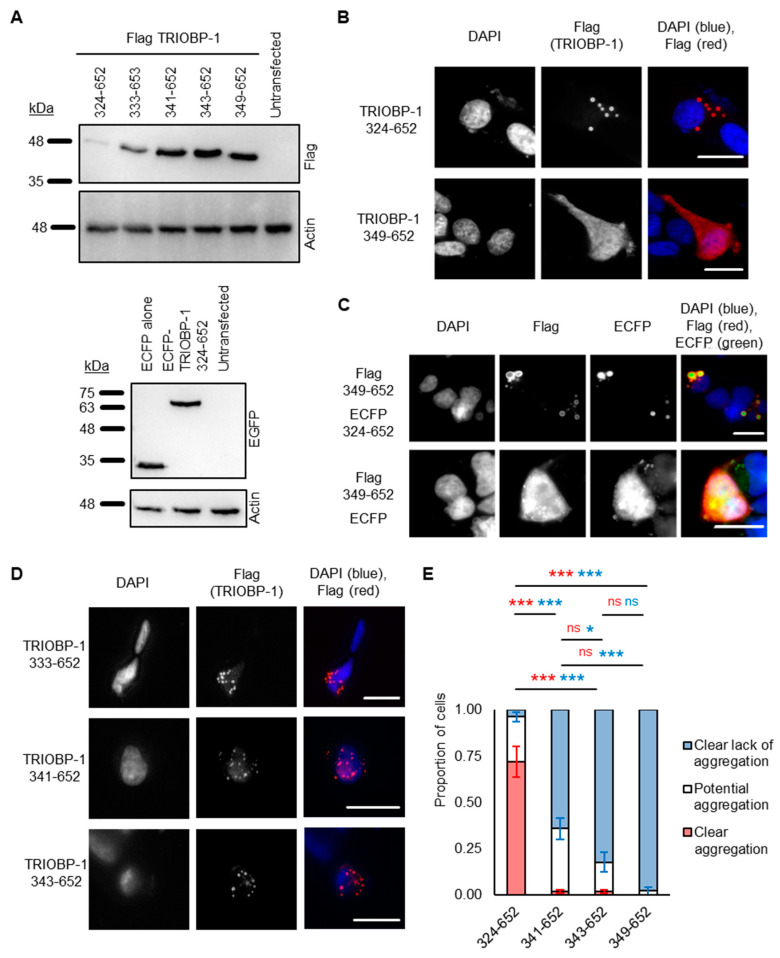
Testing aggregation of TRIOBP-1 using C-terminal fragments. (**A**) Western blots showing Flag-tagged TRIOBP-1 constructs (left) and ECFP or ECFP-fused TRIOBP-1 constructs (right) in HEK293 cell lysates. (**B**) A protein fragment containing amino acids (324-652) of TRIOBP-1 consistently forms aggregates, as expected. AA 349-652 of TRIOBP-1 consistently do not form aggregates, as expected. (**C**) When aggregating TRIOBP-1 AA 324-652 and non-aggregating TRIOBP-1 349-652 are expressed together, they co-aggregate. This does not occur due to an interaction between TRIOBP-1 349-652 and the ECFP fusion protein used. (**D**) TRIOBP-1 333-652 readily forms visible aggregates in cells, which resemble those of AA 324-652. TRIOBP-1 341-652 and 343-652 form punctate structures which are less consistent than 333-652, but may still be aggregates. (**E**) Results of a quantified, blinded experiment. AA 324-652: *n* = 171, AA 341-652: *n* = 209, AA 343-652: *n* = 185, AA 349-652: *n* = 128). For both clear aggregation (red) and clear lack of aggregation (blue): * *p*_tukey_ < 0.05, *** *p*_tukey_ < 0.001, ns: not significant. All cell photos show C-terminal fragments of TRIOBP-1 expressed in SH-SY5Y cells and are typical of 3 independent experiments. The scale bars represent 10 μm.

It was previously shown that a 25 amino acid stretch from AA 324-348 is required for aggregation in cultured cells [15]. This stretch consists of a presumed unfolded loop within the CCC domain of TRIOBP-1 and is rich in charged amino acids. To elucidate the exact amino acids responsible for its aggregation propensity, plasmids encoding N-terminally truncated human TRIOBP-1 (Figure 3A) were expressed in SH-SY5Y cells. This neuroblastoma cell line was selected for all immunofluorescent microscopy experiments, as it can be transfected with an efficiency that is plausible for these experiments (≈10%, based on immunofluorescence microscopy), but also displays neuron-like characteristics. For confirmation of plasmids by Western blotting, HEK293 cells were instead used, due to their significantly higher transfection rate and therefore higher level of protein expression.

As seen previously [15], a construct beginning at AA 324 (TRIOBP 324-652), and stretching to the C-terminus, readily formed visible aggregates in the cells, while one beginning at AA 349 (TRIOBP-1, 349-652) did not (Figure 3B). Intriguingly, however, TRIOBP-1 349-652 did form aggregates when it was co-expressed with a plasmid encoding 324-652 (Figure 3C), indicating that otherwise stable TRIOBP-1 protein can be recruited to existing TRIOBP-1 aggregates.

This previously described aggregation-critical region consists of two clusters of charged amino acids, flanking a central uncharged cluster (Figure 1B). Novel constructs were therefore generated, encoding the C-terminal half of TRIOBP-1, but only part of this 25 amino acid stretch. TRIOBP-1 333-652 was seen to readily aggregate and was indistinguishable from TRIOBP-1 324-652, indicating that the first cluster of charged amino acids is not required for aggregation (Figure 3D). This fragment was unstable, however, and expressed poorly in cells. In contrast, TRIOBP-1 341-652 and 343-652 both showed visibly reduced levels of aggregation, with aggregates visible in some cells but not others (Figure 3D). This indicates that both the uncharged region from AA 333-340 and the cluster of amino acids beginning at AA 341 are likely to be involved in the aggregation process.

To confirm this, a blinded experiment was performed in which SH-SY5Y cells were transfected with TRIOBP-1 324-652, 341-652, 343-652 and 349-652, and their aggregation status characterized and quantified. Transfected cells were then classified as either “clearly aggregating” (all visible TRIOBP-1 is present in clusters of 0.5 nm or larger), “clearly not aggregating” (TRIOBP-1 is diffuse in the cytoplasm) or “potentially aggregating” (either TRIOBP-1 is forming clusters that may or may not be aggregates, or only a small proportion of the TRIOBP-1 present resembles aggregates). TRIOBP-1 333-652 was not quantified due to a low expression level (not affected by proteasomal inhibition, Supplementary Figure S5) and very low number of surviving transfected cells, which together are indicative of protein toxicity. There was a significant effect of transfection status on aggregation (ANOVA, F > F_crit_, *p* < 0.001 for each classification). Compared to TRIOBP-1 324-652, in which obvious TRIOBP-1 aggregation occurred in approximately 70% of cells, there were essentially no fully aggregating cells seen with either TRIOBP-1 341-652 or 343-652 (Figure 3E, *p*_tukey_ < 0.001 for each). There was, however, a significant reduction in cells showing partial aggregation between TRIOBP-1 341-652 (approximately 35%) and TRIOBP-1 349-652 (less than 3%, *p*_tukey_ = 0.001). Given that the expression patterns of TRIOBP-1 324-652 and TRIOBP-1 333-652 were identical, this indicates that the uncharged region from AA 333-340 is highly involved in aggregation, while the charged AA from 341-345 is also likely to be involved.

### 2.3. Aggregation of TRIOBP-1 Can Arise through Its Isoform-Specific N-Terminal Unstructured Region

**Figure 4:**
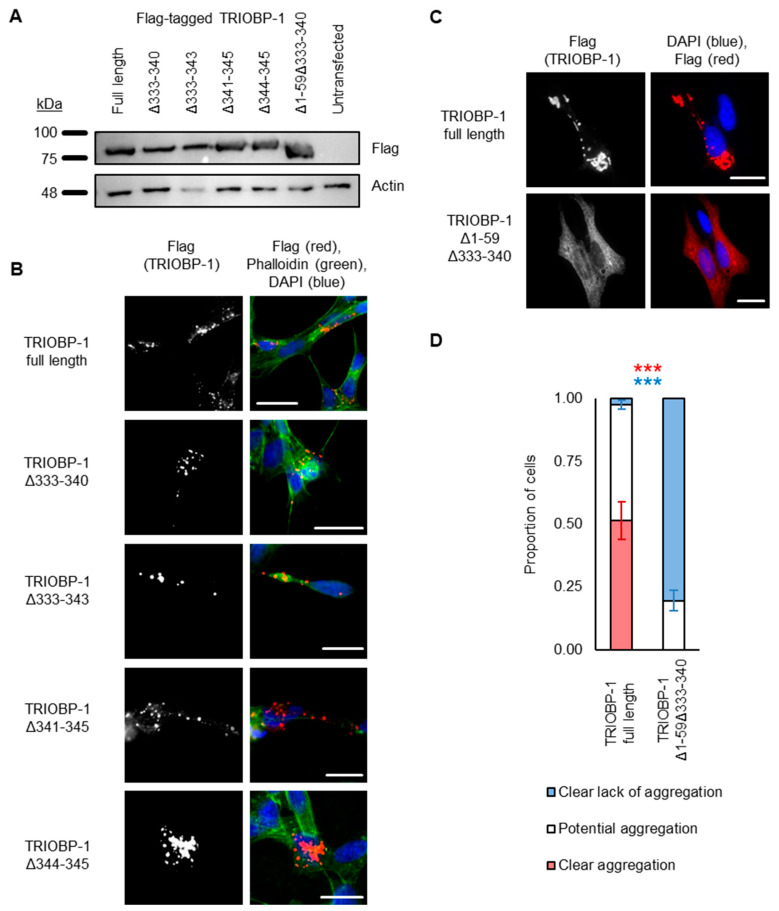
The central section of TRIOBP-1 is not the only aggregation-critical region. (**A**) Western blot of plasmids used in this figure, expressed in HEK293 cells. (**B**) Deletion of 2–11 amino acids deleted from the putative aggregation critical region in the center of the protein does not completely abolish aggregation. (**C**) Full-length TRIOBP-1 lacking the N-terminal 59 amino acid optionally translated domain and amino acids 333-340 shows almost no sign of aggregation, while the equivalent full-length protein does. (**D**) Confirmation of this difference in a blinded, quantified assay. Full length: *n* = 103 and Δ1-59Δ333-340: *n* = 101. For both clear aggregation (red) and clear lack of aggregation (blue), *** indicates *p* < 0.001. All images show Flag-tagged proteins expressed in SH-SY5Y cells (except where noted) and images are typical of 3 independent experiments. The scale bars represent 10 μm.

To confirm the importance of these residues for aggregation, constructs were generated encoding full-length TRIOBP-1, but with deletion of AA 333-340, 333-343, 341-345 or 344-345. Unexpectedly, all of these still formed aggregates when expressed in neuroblastoma cells (Figure 4A,B, with aggregation seen in the majority of transfected cells; quantified results in Supplementary Figure S6). This suggests that, while AA 333-340 are important for aggregation, they are not the only region of the protein involved. This was surprising, as previously constructs beginning immediately after the PH domain were shown not to aggregate once AA 324-348 were deleted [15]. Therefore, either the PH domain is involved in aggregation, or the N-terminal optionally translated region, which arises from two alternative start codons in TRIOBP-1 is responsible for it [15]. As previous data has suggested that the PH domain is unlikely to be involved in aggregation [9], we instead tried removing the 59 optionally translated amino acids from TRIOBP-1 (AA 1-59), in addition to deleting AA 333-340. Strikingly, this almost completely abolished aggregation compared to the full-length protein (Figure 4C), as could be confirmed in a blinded quantitative assay (Figure 4D). Specifically, introducing these deletions caused the number of cells with clearly visible aggregates to drop from around 50% to zero (*p* < 10^−3^, Welch’s *t*-test), while the number of cells with no sign of aggregation rose from less than 5% to around 80% (*p* < 10^−8^, Welch’s *t*-test). Other cells displayed TRIOBP1–1 structures that may or may not be aggregates.

### 2.4. Aggregation of TRIOBP-1 Can Occur through Either Its Optionally Translated N-Terminus, or a Loop near the Center of the Protein

**Figure 5:**
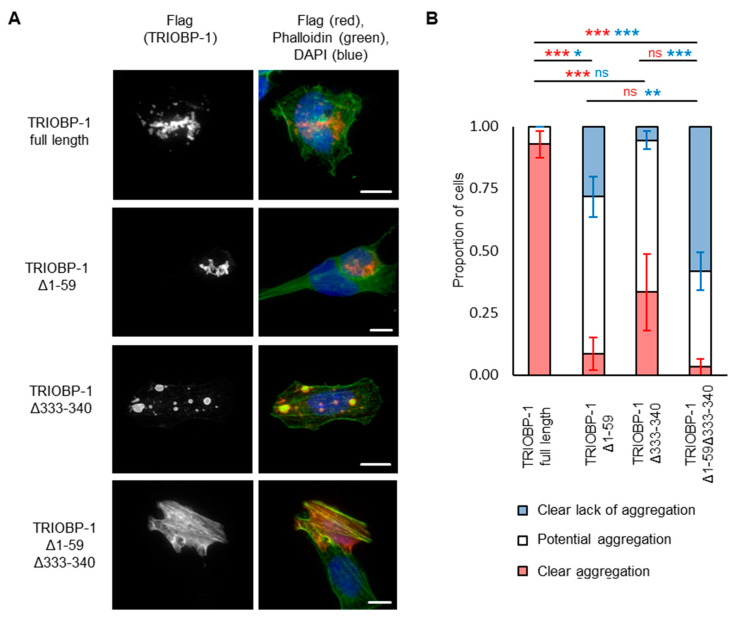
Both the extreme N-terminus and the central sections of TRIOBP-1 are involved in its aggregation, as shown through over-expression in neuroblastoma cells. (**A**) Full-length TRIOBP-1 lacking either the N-terminal 59 amino acid optionally translated domain or amino acids 333-340 show some signs of aggregation, while a double mutant lacking both does not. (**B**) Quantification of aggregation of these constructs in a blinded assay. Full length: *n* = 55 and Δ1-59: *n* = 57, Δ333-340: *n* = 30 (consistently low transfection rate), Δ1-59Δ333-340: *n* = 57. For both clear aggregation (red) and clear lack of aggregation (blue): * *p*_tukey_ < 0.05, ** *p*_tukey_ < 0.01, *** *p*_tukey_ < 0.001, ns: not significant. All images show Flag-tagged proteins are expressed in SH-SY5Y cells (except where noted) and images are typical of 3 independent experiments. The scale bars represent 10 μm.

**Figure 6:**
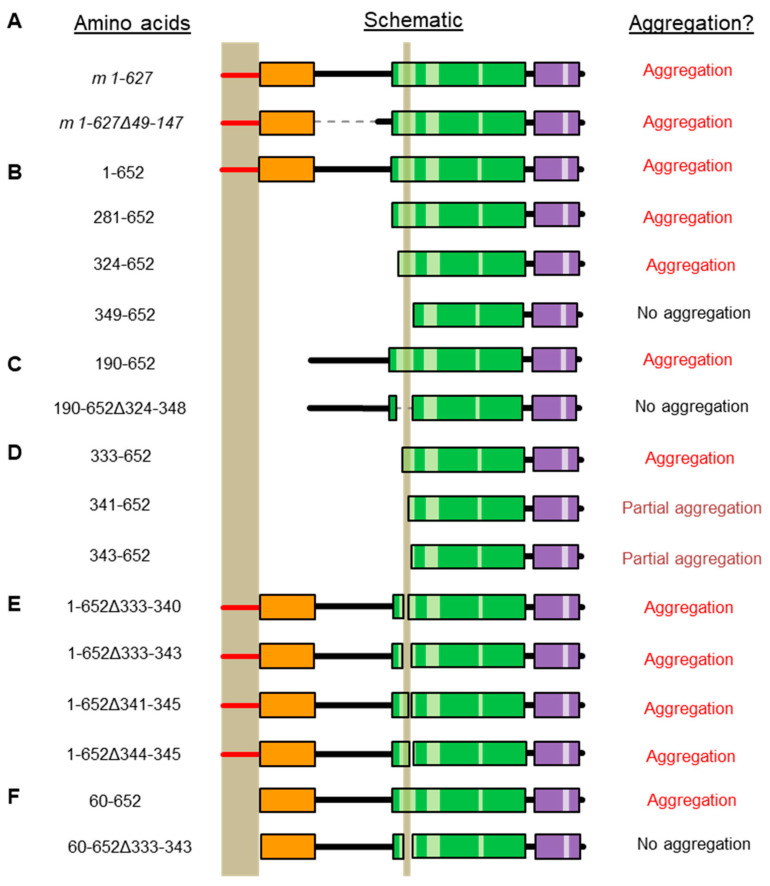
Selected TRIOBP-1 deletion constructs that have been tested for aggregation in SH-SY5Y cells and their resulting aggregation status. The domain structure of TRIOBP-1 is labeled according to Figure 1. The two putative aggregation domains are indicated as vertical bars. All vectors encode human TRIOBP-1 unless noted. (**A**) Mouse Triobp-1 constructs show the PH domain to be expendable for aggregation [9]. (**B**) N-terminal deletions of TRIOBP-1 demonstrate AA 324-328 to be essential for aggregation [15]. (**C**) Deletion of AA 324-348 in a vector lacking the optional N-terminal region and PH domain also blocks aggregation [15]. (**D**) Further N-terminally truncated vectors narrow the critical region to AA 333-340 (see Figure 3). (**E**) Deletions of the aggregation critical region in otherwise full-length TRIOBP-1 does not block aggregation (see Figure 4). (**F**) Double deletion of the N-terminal optionally translated region and the central aggregation critical region abolishes aggregation (see Figure 5).

To confirm whether the AA 333-340 of TRIOBP-1 are required for aggregation even in the absence of the optionally translated N-terminal domain, we cloned a vector encoding AA 60-652 of TRIOBP-1, corresponding to the 593 AA TRIOBP-1/Tara species. This showed signs of aggregation when expressed in neuroblastoma cells, but seemingly less than the 652 AA version (Figure 5A). In a blinded quantitative assay, it was confirmed that deletion of either AA 1-59 or AA 333-340 led to a reduction in the number of cells that showed clear TRIOBP-1 aggregation (Figure 5B, *p*_tukey_ < 0.001 and *p*_tukey_ = 0.001 respectively, ANOVA, F > F_crit_, *p* < 0.001), with deletion of AA 1-59 having the larger effect, and also showing a reduction in the number of cells that showed clear absence of TRIOBP-1 aggregation (*p*_tukey_ = 0.012, ANOVA, F > F_crit_, *p* < 0.001). Deletion of both of these regions did not significantly affect the number of clearly aggregating cells, but did increase the number of cells that showed a clear absence of aggregation (*p*_tukey_ = 0.008 and *p*_tukey_ < 0.001 when compared to deletion of AA 1-59 or 333-340 alone). Therefore, it appears that each of these regions alone can affect aggregation of TRIOBP-1, but it is the combination of the two that has the most significant effect, as summarized in Figure 6.

## 3. Discussion

The study of protein aggregation in mental illness is a relatively novel approach to uncovering the pathophysiology of these conditions, and one with the potential to act as an integration point for both genetic and environmental risk factors for them [3]. To date, several proteins have been published that may form insoluble aggregates exclusively in the brains of patients with one or more of these conditions, including TRIOBP-1, which was implicated through its insolubility in post mortem brain tissue from patients with schizophrenia [9].

**Figure 7:**
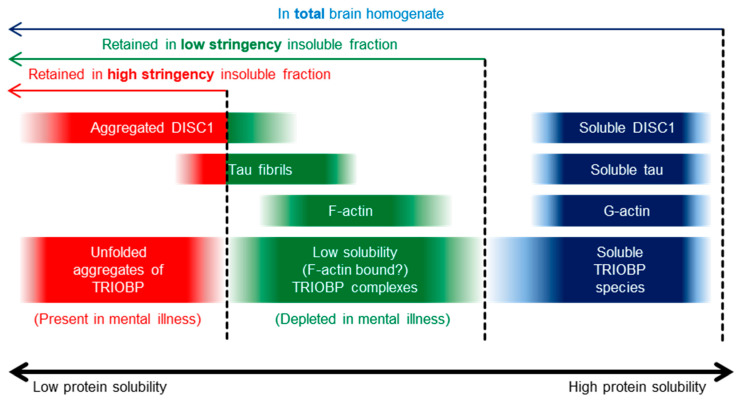
Representation of different species of TRIOBP-1, DISC1, Tau and actin described in the brain, arranged by solubility. Total brain homogenate contains all of these species, while the insoluble fractions derived from the low and high stringency purification protocols described here include only increasingly smaller subsets of insoluble protein.

We analyzed TRIOBP-1 aggregation using two insoluble protein purification protocols (Supplementary Figure S1). Of these, the low stringency protocol detects not only aggregated unfolded proteins, but also actin which is insoluble as a result of its forming high molecular weight, but still correctly folded, F-actin fibers. This protocol was originally developed as a more sensitive manner of detecting insoluble proteins, and has previously been used to identify a number of actin-related proteins as experiencing proteostatic changes in rats with cognitive impairment [25]. In comparison to the “actin-positive” low stringency protocol, the high stringency protocol is “actin-negative”, detecting aggregating transgenic DISC1, but not actin. This is the protocol, or close variations of it, that has been used in the majority of studies to date regarding protein aggregation in mental illness. Curiously, while DISC1 was present strongly in the high stringency insoluble fraction, Tau was present at a more reduced level. This is consistent with the fact that formic acid extraction, an established method for purifying insoluble Tau [28], also leads to insoluble fractions which contain actin [29], and are therefore also “actin-positive”. The combination of these two protocols, one optimized for high stringency detection of only the most insoluble aggregated proteins in the brain, and the other optimized for more sensitive detection of a wider array of insoluble protein complexes (folded or otherwise), can thus aid the separation of insoluble proteins into subtypes based either on their macromolecular structure or on whether the proteins remain folded (Figure 7).

Having previously used the high stringency protocol to determine that TRIOBP-1 is present in the pooled aggregated protein of a group of schizophrenia brain samples [9] we here demonstrate the existence of aggregated TRIOBP-1 in a broader and independent subset of patients with major mental illness. Specifically, TRIOBP-1 could be detected in the high stringency protein fraction of approximately half of the brain samples from patients examined. It is interesting to note that this occurred in both schizophrenia and major depression, suggesting that it is a mental-illness related event which crosses clinical diagnostic boundaries. This is consistent, however, with our previous identification of DISC1, dysbindin-1 and CRMP1 aggregation across multiple psychiatric diagnoses [2,7,8].

Our results suggest that TRIOBP-1 aggregation, as defined by the high stringency protocol, identifies a substantial subset of mental illness patients as sharing a common disrupted biological process, potentially more so than can be identified by DISC1 aggregation [2]. While it does not necessarily follow that aggregation of TRIOBP-1 must be a causative factor in mental illness, these data strongly imply that it forms a distinct part of the cellular pathology of these conditions. Two of the 50 samples from control individuals also displayed aggregated TRIOBP-1. These may be false positives; however, the presence of aggregated TRIOBP-1 in two chronic mental illnesses also suggests the possibility that it in fact exists in a broader range of conditions, and it thus cannot be discounted that these two control individuals may have carried an unreported, presumably subtler, condition which is also associated with this aggregation event. Replication of our findings in even bigger patient samples, ideally with differing ethnicities, would therefore prove revealing.

In comparison, when the low stringency protein purification technique was employed on the brain samples, we observed an inverse correlation of the amount of pelleted material with that of the high stringency protocol. Since the low stringency protocol also purifies F-actin, it is likely that the TRIOBP species detected in this fraction are present due to their direct association with F-actin, as part of the known role of TRIOBP-1 in actin polymerization [11,14,15]. In contrast to findings with the high stringency protocol, the abundance of these TRIOBP species is actually decreased in mental illness. It is therefore possible that their actin-binding function is inhibited, either as a cause or as a consequence of biological changes involved in mental illness.

When TRIOBP-1 was first found to aggregate in mental illness, bioinformatic analyses were performed to predict whether certain regions of the protein may have the propensity to induce aggregation. This implicated the PH domain, although deletion of this region did not affect aggregation of mouse TRIOBP-1 [9] (Figure 6A). Subsequent studies involved expressing only the C-terminal half of the protein, consisting of most of the coiled-coil domains, but lacking the first coiled-coil and/or the loop between the first two coiled-coils. These experiments confirmed that this loop, mapped down to 25 amino acids (AA 324-348), was critical for aggregation [15] (Figure 6B). Crucially, a longer construct incorporating the unstructured “mid-region” of TRIOBP-1 and the first coiled-coil, but lacking these 25 amino acids, still did not form aggregates [15] (Figure 6C). Here we began by also using C-terminal fragments of TRIOBP-1 including only a portion of the 25 amino acid-long loop between the first two coiled-coils, allowing us to narrow down the likely aggregation domain to just 8 amino acids (AA 333-340, Figure 6D). We, therefore, anticipated that when these amino acids were deleted from otherwise full-length TRIOBP-1, the aggregation propensity of the process would be abolished, as was seen with a longer construct previously (Figure 6C). Surprisingly, however, knocking out of these 8 amino acids, or other portions of the loop, had no obvious effect on TRIOBP-1 aggregation (Figure 6E), indicating that the optionally translated N-terminal region of TRIOBP-1 may also be able to induce aggregation. This was confirmed by the deletion of both the N-terminal untranslated region (AA 1-59) and the 8 amino acids from between coiled-coils 1 and (AA 333-340), which together abolished aggregation (Figure 6F). It is therefore apparent that either of these regions alone is capable of inducing aggregation of TRIOBP-1. However, based on assays knocking out one or both sites, it is clear that aggregation occurs most consistently when both sites are present in the protein.

While the 652 AA long form of TRIOBP-1 therefore seemingly has two mechanisms through which it can form aggregates, one based on its extreme N-terminus, and one based on the loop between two coiled-coils in the center of the protein, it should be noted that this form of TRIOBP-1 likely represents a minority of TRIOBP-1 protein species in the cell [15]. The presence of a second Kozak sequence and start codon means that TRIOBP-1 can also be expressed as a 597 AA long protein, starting at AA 60, as counted in this paper. Indeed, many studies, both classic and recent, assume that this is the principle TRIOBP-1 species. 597 AA long TRIOBP-1, referred to as Tara in many publications, therefore lacks the N-terminal unstructured region, and possesses only one aggregation-critical region, implying only one mechanism of aggregation. Indeed, it is possible that the doublet of TRIOBP bands seen around 72 kDa in the brain samples represents the 652 and 597 AA long forms of TRIOBP-1, although other possibilities, including protein phosphorylation [19,22,23,30], could also account for it. Similarly, longer splice variants of TRIOBP, such as TRIOBP-5 and TRIOBP-6 should also form protein aggregates [15,17], presumably as a result of them having extensive sequences overlapping with TRIOBP-1. While these long TRIOBP isoforms contain the central aggregation region of TRIOBP-1, they lack the N-terminal 59 AA unstructured region [12,13,14], again suggesting that their aggregation can occur only through the central aggregation region and its associated mechanism. Finally, smaller TRIOBP species of 40-60 kDa were detected in insoluble fractions of brain samples here, and were also seen previously in cell systems [9], which are detectable using antibodies against C-terminal parts of TRIOBP-1. While it is unclear whether these species represent the products of additional TRIOBP splice variants or processed forms of TRIOBP-1, it is likely that they would also possess only the one, central, aggregation critical region.

Determining mechanistic details of the two paths by which TRIOBP can form protein aggregates, therefore, represents an important next step in investigating this emerging aspect of mental illness molecular pathology. Additionally, the existence of plasmid constructs encoding near-full-length TRIOBP-1 that does not aggregate provides a powerful tool for future experiments to investigate the consequences of aggregation. For example, while rodent models of DISC1 and dysbindin-1 aggregation show clear effects on behavior, brain anatomy and biochemistry [27,31,32], it remains to be confirmed to what extent these phenotypes arise from the presence of human aggregation protein in the brain, and how much from over-expression of the human protein in the brain at all. This could be addressed, to a significant extent, in a system involving three lines of animals, one control lacking a transgene, one expressing full-length aggregating protein, and one expressing a mutant version of the protein that lacks or has a significantly reduced tendency to aggregate, such as the TRIOBP-1 lacking amino acids 1-59 and 333-340.

Uncovering the aggregation critical regions of TRIOBP, therefore, gives a powerful experimental tool to help understand the mechanisms behind this potentially pathological event, which is now implicated in the pathology of both schizophrenia and major depressive disorder, but also to understand the consequences of that aggregation for mental illness.

## 4. Materials and Methods

### 4.1. Brain Samples

Post mortem human brain specimens from BA23, the anterior cingulate cortex, were obtained from 50 control individuals, 25 patients with schizophrenia and 25 patients with major depressive disorder from the Human Brain and Tissue Repository of the Lieber Institute for Brain Development. BA23 was dissected under visual guidance using a hand-held dental drill, just superior to the rostrum of the corpus callosum. Details of the informed consent, diagnostic procedures, and curation are elaborated in detail in a previous publication from our group [33]. Demographic details of these can be found in Supplementary Table S1. Brains from a transgenic rat expressing human DISC1 [27] were extracted according to methods approved by the LANUV (State Agency for Nature, Environment and Consumer Protection), North Rhine-Westphalia, Germany. Brains from mice over-expressing a 383 AA form of human Tau with a P301S mutation [26] were a gift from Dr. Michel Goedert (University of Cambridge, Cambridge, UK).

### 4.2. High Stringency Insoluble Protein Fraction Purification Protocol

Brain samples were homogenized to 10% (*w*/*v*) in 50 mM HEPES pH 7.5/250 mM sucrose/5 mM magnesium chloride/100 mM potassium acetate/2 mM PMSF containing protease inhibitor cocktail, and were lysed by the addition of Triton X-100 to a final concentration of 0.5% (*v*/*v*). Lysate was then centrifuged at 20,000× *g* for 20 min. Pellet was resuspended in the same buffer (including Triton X-100) and centrifuged a second time. Pellet was resuspended in 50 mM HEPES pH 7.5/1.6 M sucrose/100 mM potassium acetate/1 mM PMSF/0.5% Triton X-100 and ultracentrifuged at 130,000× *g* for 45 min. This was repeated a second time. Pellet was resuspended in 50 mM HEPES pH 7.5/1 M sodium chloride/20 mM magnesium chloride/30 mM calcium chloride containing 40 units/mL DNaseI and protease inhibitor cocktail. Lysate was then incubated at 4 °C for 16 h on a rotary wheel. Lysate was ultracentrifuged at 130,000× *g* for 45 min. Pellet was resuspended in this same buffer (minus the DNaseI) and ultracentrifuged at 130,000× *g* for 45 min. Pellet was resuspended in 50 mM HEPES pH7.5/0.5% sarkosyl and ultracentrifuged at 130,000× *g* for 45 min. This was repeated, and the final pellet was resuspended in dissolution buffer (10 mM Tris/0.5 M triethylammonium bicarbonate/8 M urea/1% SDS). This entire procedure, including centrifugation steps, was done at 0–4 °C, is adapted and optimized from one of our previously published protocols [2] and is summarized in Supplementary Figure S1.

### 4.3. Low Stringency Insoluble Protein Fraction Purification Protocol

Brain samples were homogenized to 10% (*w*/*v*) in 50 mM HEPES pH 7.5/250 mM sucrose/5 mM magnesium chloride/100 mM potassium acetate/2 mM PMSF containing protease inhibitor cocktail. Homogenates were lysed by the addition of lysis buffer (in a 2:1 homogenate to buffer ratio): 50 mM HEPES pH 7.5/250 mM sucrose/20 mM magnesium chloride/100 mM potassium acetate/2 mM PMSF/3% NP-40/0.6% sarkosyl/15 mM glutathione/120 units/mL DNaseI, containing protease inhibitor cocktail. Lysis was performed overnight at 4 °C on a rotary wheel. The following buffer was applied in a 5:4 (lysate to buffer) ratio: 50 mM HEPES pH 7.5/2.3 M sucrose/5 mM glutathione/1 mM PMSF/1% NP-40/0.2% sarkosyl. Lysate was then ultracentrifuged at 100,000× *g* for 45 min. Pellet was resuspended in 50 mM HEPES pH 7.5/1.6 M sucrose/5 mM glutathione/1 mM PMSF/1% NP-40/0.2% sarkosyl and ultracentrifuged at 100,000× *g* for 45 min. Pellet was resuspended in 50 mM HEPES pH 7.5/1.5 M NaCl/5 mM glutathione and ultracentrifuged at 100,000× *g* for 45 min. Pellet was resuspended in 50 mM HEPES pH 7.5/250 mM sucrose/5 mM glutathione/1% NP-40/0.2% sarkosyl and ultracentrifuged at 100,000× *g* for 45 min. Final pellet was resuspended in dissolution buffer. This entire procedure, including centrifugation steps, was done at 0–4 °C, is adapted and optimized from one of our previously published protocols [25] and is summarized in Supplementary Figure S1.

### 4.4. Plasmids

Plasmids encoding full-length TRIOBP-1 and two N-terminally truncated versions of it, have been described previously [15]. Additional constructs encoding the coiled-coil regions of TRIOBP-1 were made by subcloning reading frames, and then transferring into either a Gateway entry vector, either pDONR/Zeo (using BP clonase recombination, enzyme and plasmids: Thermo Fisher Scientific, Waltham, NJ, USA) or pENTR1A no ccDB (Dr. Eric Campeau, supplied by AddGene, Watertown, MA, USA, clone 17398 [34], by ligation). Vectors encoding full-length TRIOBP-1 with internal deletions were produced by subcloning of 5′ and 3′ fragments of the gene, which were then sequentially ligated into pENTRA1A no ccDB. Reading frames were transferred from entry vectors into destination vectors using LR clonase II recombination (Thermo Fisher Scientific). Destination vectors used were pdcDNA-FlagMyc (B. Janssens, supplied by the BCCM/LMBP Plasmid Collection, Zwijnaarde, Belgium, clone LMBP 4705) and pdECFP (Dr. S. Wiemann, BCCM/LMBP Plasmid Collection, clone LMBP 4548 [35]). All vectors were confirmed by sequencing. Details of all plasmids used are in Supplementary Table S2 and the primers used to clone them are in Supplementary Table S3.

### 4.5. Antibodies

Commercial primary antibodies were purchased against actin (Origene, Rockville, MD, USA, TA811000S, Merck, Darmstadt, Germany, A2066), Flag tag (Merck, F1804), total and phosphorylated tau (Thermo Fischer Scientific, MN1000, MN1020), TRIOBP-1 (Atlas Antibodies, Bromma, Sweden, HPA003747) and tubulin (Merck, T9026). Monoclonal antibody 14F2 against human DISC1 has been described previously [7]. Secondary antibodies were purchased from Thermo Fisher Scientific (A11005, A32723, 31430) and LI-COR Biosciences, Lincoln, NE, USA, 926-68071).

### 4.6. Cell Culture

Human SH-SY5Y neuroblastoma cells were cultured in D-MEM/F-12 with 10% fetal calf serum (both Thermo Fisher Scientific), 1× MEM non-essential amino acids, penicillin and streptomycin (all Pan Biotech, Aidenbach, Germany). Human HEK293 kidney cells were cultured in D-MEM (Thermo Fisher Scientific) with 10% Cosmic Calf Serum (Cytiva, Marlborough, MA, USA), penicillin and streptomycin (Pan Biotech). Plasmids transfections were performed using Metafectene (Biontex, Munich, Germany, for HEK293), Metafectene Pro (Biontex, for some SH-SY5Y experiments) or Lipofectamine 2000 (Thermo Fischer Scientific, for some SH-SY5Y experiments), according to the manufacturer’s instructions. For one experiment, the proteasomal inhibitor MG132 (MedChemExpress, Monmouth Junction, NJ, USA) was added to 10 μM for 16 h after transfection, with DMSO used as a vehicle control.

### 4.7. Western Blotting

Cell lysates were run on Bis-acrylamide gels or NuPAGE Bis-Tris gels (Thermo Fisher Scientific), and transferred to membranes using a Trans-Blot Turbo Transfer System (Bio-Rad Laboratories, Hercules, CA, USA). Membranes were blocked for one hour with PBS/0.5% Tween 20/5% skimmed milk powder and then stained with primary and secondary antibodies. Protein was visualized either using ECL Western Blotting Substrate (Thermo Fischer Scientific) on a ChemiDoc MP Imaging System (Bio-Rad) with ImageLab software (Bio-Rad), using ECL and X-ray development, or on an Odyssey Clx infrared imaging system and associated analysis software (LI-COR Biosciences).

### 4.8. Immunocytochemistry and Microscopy

Cells were seeded on glass coverslips, before being fixed and permeabilized with PBS/4% paraformaldehyde (15 min) and PBS/0.5% Triton X-100 (10 min), respectively. Cells were blocked with PBS/10% goat serum and then stained with primary and secondary antibodies. Coverslips were mounted using 90% glycerol/100 mM Tris pH 8.8/0.5% propyl gallate or Fluoroshield histology stain (Merck) and then viewed on an IX83 fluorescent microscope (Olympus, Tokyo, Japan) with Orca R2 CCD camera (Hamamatsu Photonics, Hamamatsu City, Japan). CellSens software was used (Olympus). Nuclei were visualized with DAPI (Merck) and F-actin with Acti-stain 488 phalloidin (Cytoskeleton, Denver, CO, USA). All images displayed are typical of at least three independent experiments. For quantified experiments, samples were encoded, so that transfections, staining, image collection and aggregation analysis could be conducted by a researcher blinded to construct status. Data was then decoded for statistical analysis using JASP (version 0.14.1, Amsterdam, The Netherlands) [36]. Six coverslips of cells were transfected with each plasmid, and the number of cells containing each aggregation status was then averaged over the coverslips. When comparing four groups, ANOVA was used with Brown–Forsythe correction and Tukey’s post hoc test, while for comparing two groups, data was analyzed using Welch’s *t*-test.

## Supplementary Materials

The supporting information can be downloaded at: https://www.mdpi.com/article/10.3390/ijms231911048/s1.
