# Supplementary material for: TRIOBP-1 Protein Aggregation Exists in Both Major Depressive Disorder and Schizophrenia, and Can Occur through Two Distinct Regions of the Protein
Source: Int J Mol Sci. 2022 Sep 21;23(19):11048. doi: 10.3390/ijms231911048 (PMC9569677; doi:10.3390/ijms231911048)
Supplement: Supplementary file 1 [file ijms-23-11048-s001.zip › ijms-1879249-supplementary.pdf]

# TRIOBP-1 Protein Aggregation Exists in Both Major Depressive Disorder and Schizophrenia, and Can Occur through Two Distinct Regions of the Protein

Beti Zaharija, Maja Odorčić, Anja Hart, Bobana Samardžija, Rita Marreiros,

Ingrid Prikulis, Maja Juković, Thomas M. Hyde, Joel E. Kleinman,

Carsten Korth and Nicholas J. Bradshaw

**Supplementary material**

Supplementary tables S1-S3

Supplementary figures S1-S6

| | | Control | Schizophrenia | Major depression |
|---|---|---|---|---|
| **Race** | African American | 78 | 68 | 36 |
| (%) | Asian American | 4 | 0 | 0 |
| | Caucasian American | 16 | 28 | 64 |
| | Hispanic American | 2 | 4 | 0 |
| **Gender** | Male | 66 | 40 | 44 |
| (%) | Female | 34 | 60 | 56 |
| **Age** | (mean) | 45.4 | 53.5 | 47.7 |
| **pH** | (mean) | 6.52 | 6.38 | 6.20 |
| **PMI** | (mean) | 32.6 | 40.4 | 41.5 |

**Supplemental table S1**: Demographic information regarding the donors of the brain samples used in this manuscript. In total there were samples from 50 control individuals, 25 schizophrenia patients and 25 major depressive disorder patients.

| No. | Vector backbone | Gene insert | Origin |
|---|---|---|---|
| 1 | pDONR Zeo | (None) | Thermo Fisher Scientific |
| 2 | pENTR1A no ccDB | (None) | Addgene, clone 17398, Campeau et al (2009) PLOS One 4:e6529 |
| 3 | pdcDNA-FlagMyc | (None) | BCCM/LMBP Plasmid Collection, clone LMBP 4705 |
| 4 | pdECFP | (None) | BCCM/LMBP Plasmid Collection, clone LMBP 4548 |
| 5 | pENTR1A | TRIOBP-1 full length | Bradshaw et al (2017) J. Biol. Chem. 292:9583-9598 |
| 6 | pDONR/Zeo | TRIOBP-1 (333-652) | Subcloned from plasmid 5 using primers B & E, then A & E Then BP clonase recombination into plasmid 1. |
| 7 | pDONR/Zeo | TRIOBP-1 (341-652) | Subcloned from plasmid 5 using primers C & E, then A & E. Then BP clonase recombination into plasmid 1. |
| 8 | pDONR/Zeo | TRIOBP-1 (343-652) | Subcloned from plasmid 5 using primers D & E, then A & E. Then BP clonase recombination into plasmid 1. |
| 9 | pDONR/Zeo | TRIOBP-1 (349-652) | Bradshaw et al (2017) J. Biol. Chem. 292:9583-9598 |
| 10 | pENTR1A | TRIOBP-1 (1-332) | Subcloned from plasmid 5 using primers F and H. Restriction digested and ligated into the *SalI* and *EcoRI* sites of plasmid 2. |
| 11 | pENTR1A | TRIOBP-1 (1-340) | Subcloned from plasmid 5 using primers F and I. Restriction digested and ligated into the *SalI* and *EcoRI* sites of plasmid 2. |
| 12 | pENTR1A | TRIOBP-1 (1-343) | Subcloned from plasmid 5 using primers F and J. Restriction digested and ligated into the *SalI* and *EcoRI* sites of plasmid 2. |
| 13 | pENTR1A | TRIOBP-1 (1-332,341-652) | Subcloned from plasmid 5 using primers K and L. Restriction digested and ligated into the *KpnI* and *XbaI* sites of plasmid 10. |
| 14 | pENTR1A | TRIOBP-1 (1-332,344-652) | Subcloned from plasmid 5 using primers K and M. Restriction digested and ligated into the *KpnI* and *XbaI* sites of plasmid 10. |
| 15 | pENTR1A | TRIOBP-1 (1-340,346-652) | Subcloned from plasmid 5 using primers K and N. Restriction digested and ligated into the *KpnI* and *XbaI* sites of plasmid 11. |
| 16 | pENTR1A | TRIOBP-1 (1-343,346-652) | Subcloned from plasmid 5 using primers K and N. Restriction digested and ligated into the *KpnI* and *XbaI* sites of plasmid 12. |
| 17 | pENTR1A | TRIOBP-1 (60-652) | Subcloned from plasmid 5 using primers G and K. Restriction digested and ligated into the *SalI* and *XbaI* sites of plasmid 2. |

| No. | Vector backbone | Gene insert | Origin |
|---|---|---|---|
| 18 | pENTR1A | TRIOBP-1 (60-332,341-652) | Subcloned from plasmid 13 using primers G and K. Restriction digested and ligated into the *SalI* and *XbaI* sites of plasmid 2. |
| 19 | pdcDNA-Flag | TRIOBP-1 full length | Bradshaw et al (2017) J. Biol. Chem. 292:9583-9598 |
| 20 | pdcDNA-Flag | TRIOBP-1 (324-652) | Bradshaw et al (2017) J. Biol. Chem. 292:9583-9598 |
| 21 | pdcDNA-Flag | TRIOBP-1 (333-652) | LR clonase recombination of plasmids 3 and 6 |
| 22 | pdcDNA-Flag | TRIOBP-1 (341-652) | LR clonase recombination of plasmids 3 and 7 |
| 23 | pdcDNA-Flag | TRIOBP-1 (343-652) | LR clonase recombination of plasmids 3 and 8 |
| 24 | pdcDNA-Flag | TRIOBP-1 (349-652) | Bradshaw et al (2017) J. Biol. Chem. 292:9583-9598 |
| 25 | pdcDNA-Flag | TRIOBP-1 (1-332,341-652) | LR clonase recombination of plasmids 3 and 13 |
| 26 | pdcDNA-Flag | TRIOBP-1 (1-332,344-652) | LR clonase recombination of plasmids 3 and 14 |
| 27 | pdcDNA-Flag | TRIOBP-1 (1-340,346-652) | LR clonase recombination of plasmids 3 and 15 |
| 28 | pdcDNA-Flag | TRIOBP-1 (1-343,346-652) | LR clonase recombination of plasmids 3 and 16 |
| 29 | pdcDNA-Flag | TRIOBP-1 (60-652) | LR clonase recombination of plasmids 3 and 17 |
| 30 | pdcDNA-Flag | TRIOBP-1 (60-332,341-652) | LR clonase recombination of plasmids 3 and 18 |
| 31 | pdECFP | Control | LR clonase recombination of plasmids 2 and 4 |
| 32 | pdECFP | TRIOBP-1 (349-652) | LR clonase recombination of plasmids 4 and 9 |

**Supplemental table S2**: Details of plasmids used in this manuscript. For references to primers, see supplementary table S3.

| Code | Name | Primer |
|---|---|---|
| A | Ext attB1 F | GTCGACACAAGTTTGTACAAAAAAGCAGGCTTCGCCGCCACC |
| B | 333 attB1 F | AAGCAGGCTTCGCCGCCACCATGCTCACTGCCCTGCTCAAC |
| C | 341 attB1 F | AAGCAGGCTTCGCCGCCACCATGCGCGGAGAGCGCCG |
| D | 343 attB1 F | AAGCAGGCTTCGCCGCCACCATGGAGCGCCGAGGGC |
| E | 652 attB2 R | AAGCAGGCTTCGCCGCCACCATGCTCACTGCCCTGCTCAAC |
| F | 1 SalI F | GATTAGTCGACATGGGCGGATGGAAG |
| G | 60 SalI F | GGATAGTCGACATGACGCCCGATC |
| H | 332 kpnIecoRI R | GATTAGAATTCAGGTACCGGGCACCCGC |
| I | 340 kpnIecoRI R | GGTTGGAATTCAGGTACCGCTTTGGTTG |
| J | 343 kpnIecoRI R | GATTAGAATTCAGGTACCCTCTCCGCGG |
| K | 652 xbaI R | GTGTTTCTAGATCACTACTCAGCCAG |
| L | 341 kpnI F | GTTGGTACCCGCGGAGAGC |
| M | 344 kpnI F | GTATTGGTACCCGCCGAGGGC |
| N | 346 kpnI F | GTATTGGTACCGGGCCCCCAAG |

**Supplemental table S3**: Details of primers used to clone the plasmids in supplementary table S2

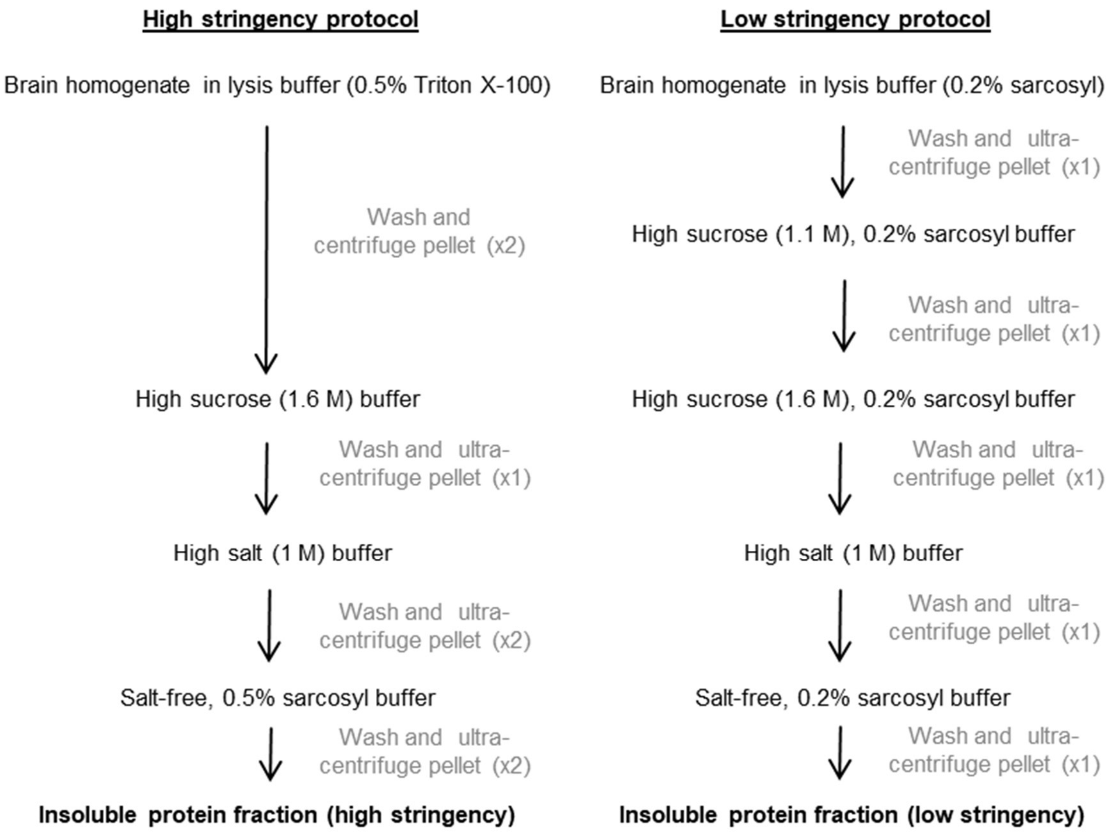


**Supplemental figure S1:** The high and low stringency insoluble protein purification protocols. Flow charts summarizing the steps required in purifying the total insoluble protein fractions of brain material using the high and low stringency purification protocols respectively. Full detail can be found in the Materials and Methods section

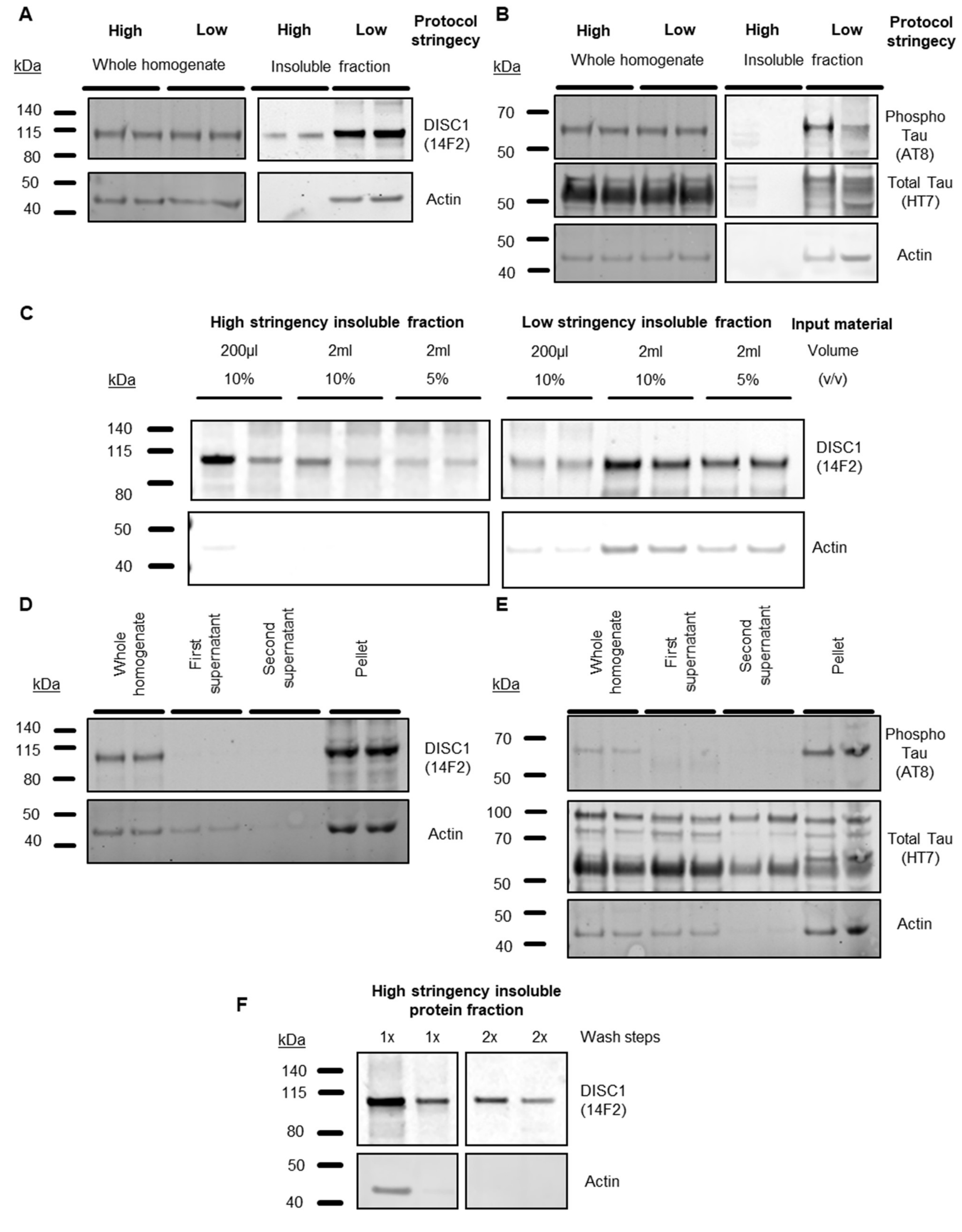
A
High
Low
High
Low
Protocol stringecy
kDa
Whole homogenate
Insoluble fraction
140
115
80
50
40
DISC1 (14F2)
Actin
B
High
Low
High
Low
Protocol stringecy
kDa
Whole homogenate
Insoluble fraction
70
50
50
50
40
Phospho Tau (AT8)
Total Tau (HT7)
Actin
C
High stringency insoluble fraction
Low stringency insoluble fraction
Input material
200µl
2ml
2ml
200µl
2ml
2ml
Volume
10%
10%
5%
10%
10%
5%
(v/v)
kDa
140
115
80
50
40
DISC1 (14F2)
Actin
D
Whole homogenate
First supernatant
Second supernatant
Pellet
kDa
140
115
80
50
40
DISC1 (14F2)
Actin
E
Whole homogenate
First supernatant
Second supernatant
Pellet
kDa
70
50
100
70
50
50
40
Phospho Tau (AT8)
Total Tau (HT7)
Actin
F
High stringency insoluble protein fraction
kDa
1x
1x
2x
2x
Wash steps
140
115
80
50
40
DISC1 (14F2)
Actin

**Supplemental figure S2** (previous page). Analysis and standardisation of the high and low stringency insoluble protein purification protocols. (A) Blots showing the purified insoluble fractions of protein from 3-month old DISC1 transgenic rat brain[1], demonstrating aggregated DISC1 to be detectible using both purification protocols, while insoluble but folded F-actin is detected only using the mild protocol. (B) Equivalent blot looking at Tau and actin in the brain of 6-month old TauP301S mice[2]. For ease of viewing, insoluble fractions are enriched relative to the whole homogenate in these images (high stringency protocol: 6.67-fold, low stringency: 10-fold). (C) The effect of using different amounts of brain homogenate starting material in the two purification approaches, demonstrated using DISC1 transgenic rat brain homogenate. (D) The lower amount of final insoluble protein yielded by the high stringency protocol is not solely as a result of the low speed centrifugation step at the beginning of the process. When this centrifugation is performed twice on the crude brain homogenate of 3-month old DISC1 transgenic rat, some DISC1 is lost in the supernatant, but the majority is retained in the ensuing pellet. (E) Equivalent test performed on 6-month old TauP301S mice, showing a loss of total Tau into the supernatants, but with phosphorylated Tau, which would be expected to be insoluble, being largely retained in the pellet. (F) The importance of including replicate wash steps in the high stringency protein purification protocol is demonstrated by parallel purifications of such protein from DISC1 transgenic rat brain (200µl 5% homogenate) including each wash step only once (“1x”) or in duplicate (“2x”). When only one wash is included, greater amounts of DISC1 are seen in the purified faction, along with insoluble, but still folded, F-actin.

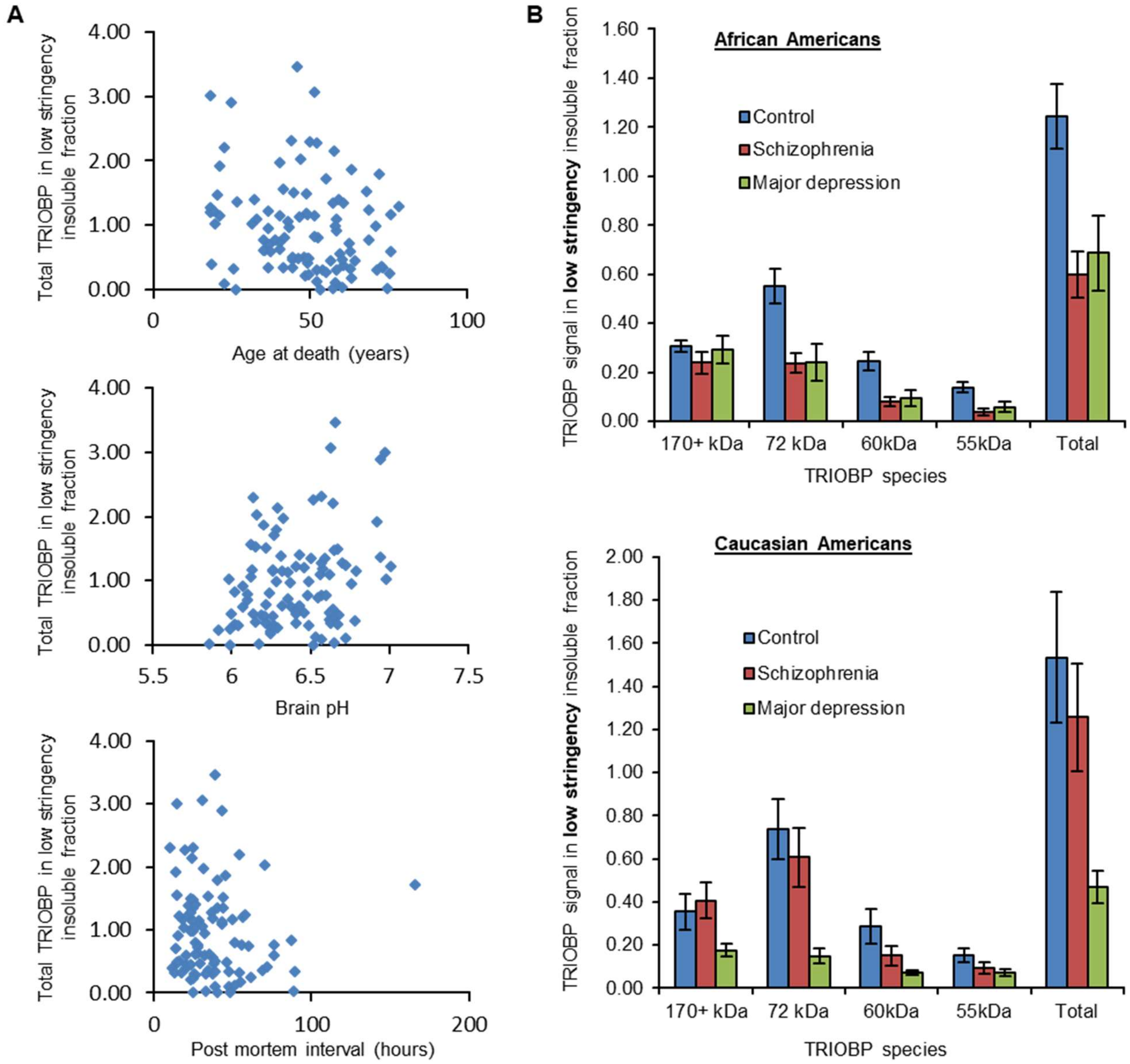


**Supplemental figure S3:** Effect of demographic factors on the amount of TRIOBP in the low stringency insoluble protein fraction. (A) Comparison of the total amount of TRIOBP (total signal of all four species in the Western blot) in the low stringency insoluble protein fraction with age of death, pH or post mortem interval of the brain samples. The only significant correlation is with brain pH (Pearson correlation coefficient 0.249, p = 0.012). (B) Quantified insoluble TRIOBP, defined using the low stringency protocol, in the brain samples from each of the control, schizophrenia and major depression groups. Data is split between the two major ethnicities present in the sample (Asian American and Hispanic samples excluding due to the low number present). Alterations in insoluble TRIOBP-1 (low stringency protocol) in schizophrenia may be more pronounced in African Americans in this sample than in Caucasian Americans, with the inverse seemingly being true for some TRIOBP-1 species in major depression.

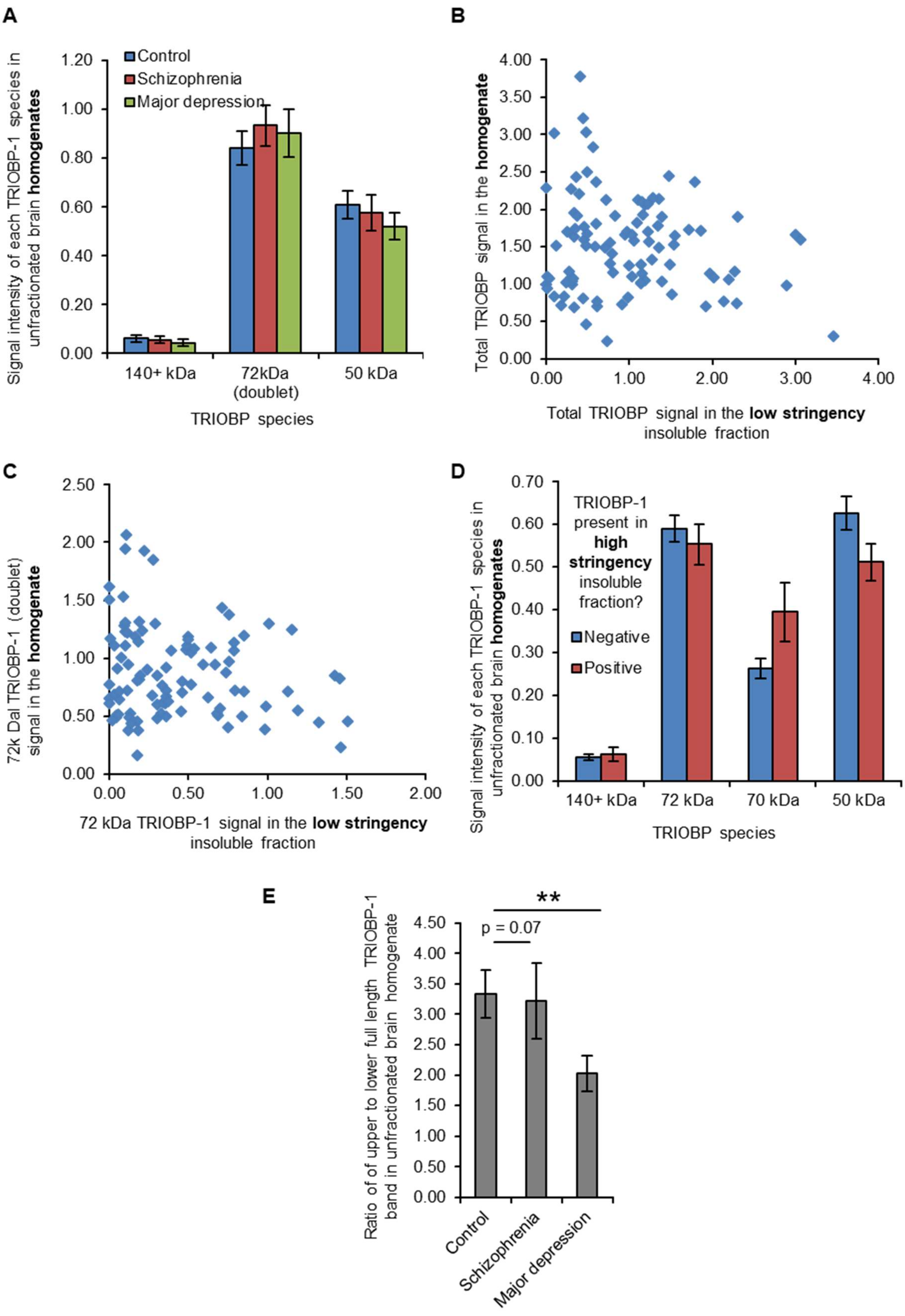
A
Signal intensity of each TRIOBP-1 species in unfractionated brain homogenates
Control
Schizophrenia
Major depression
140+ kDa
72kDa (doublet)
50 kDa
TRIOBP species
B
Total TRIOBP signal in the homogenate
Total TRIOBP signal in the low stringency insoluble fraction
C
72k Dal TRIOBP-1 (doublet) signal in the homogenate
72 kDa TRIOBP-1 signal in the low stringency insoluble fraction
D
Signal intensity of each TRIOBP-1 species in unfractionated brain homogenates
TRIOBP-1 present in high stringency insoluble fraction?
Negative
Positive
140+ kDa
72 kDa
70 kDa
50 kDa
TRIOBP species
E
Ratio of of upper to lower full length TRIOBP-1 band in unfractionated brain homogenate
**
p = 0.07
Control
Schizophrenia
Major depression

**Supplemental figure S4** (previous page): The relationship between levels of TRIOBP in the high and low stringency insoluble protein fractions. (A) The level of the TRIOBP-1 species present in unfractionated brain homogenate is not associated with major mental illness in these tests. Note that the 40 kDa species seen in the low stringency insoluble fraction could not be readily detected and quantified due to low abundance. (B) No correlation between levels of TRIOBP (all species) in the brain homogenate and levels of TRIOBP (all species) in the low stringency insoluble fraction. (C) Similar lack of correlation when only the full length TRIOBP-1 species (approximately 72 kDa) are considered. (D) Levels of most TRIOBP species in the unfractionated brain homogenate do not correlated with the presence of TRIOBP-1 in the high stringency insoluble fraction, although the lower of the two full length TRIOBP-1 species (styled here as “70 kDa”) shows a non-significant trend towards being more abundant in brain homogenates which also contained TRIOBP-1 in the high stringency insoluble fraction. (E) The ratio between the higher and lower full length TRIOBP-1 species in unfractionated brain homogenate (approximately 72 kDa) is statistically associated with major depression, but not schizophrenia. **: $p < 0.01$.

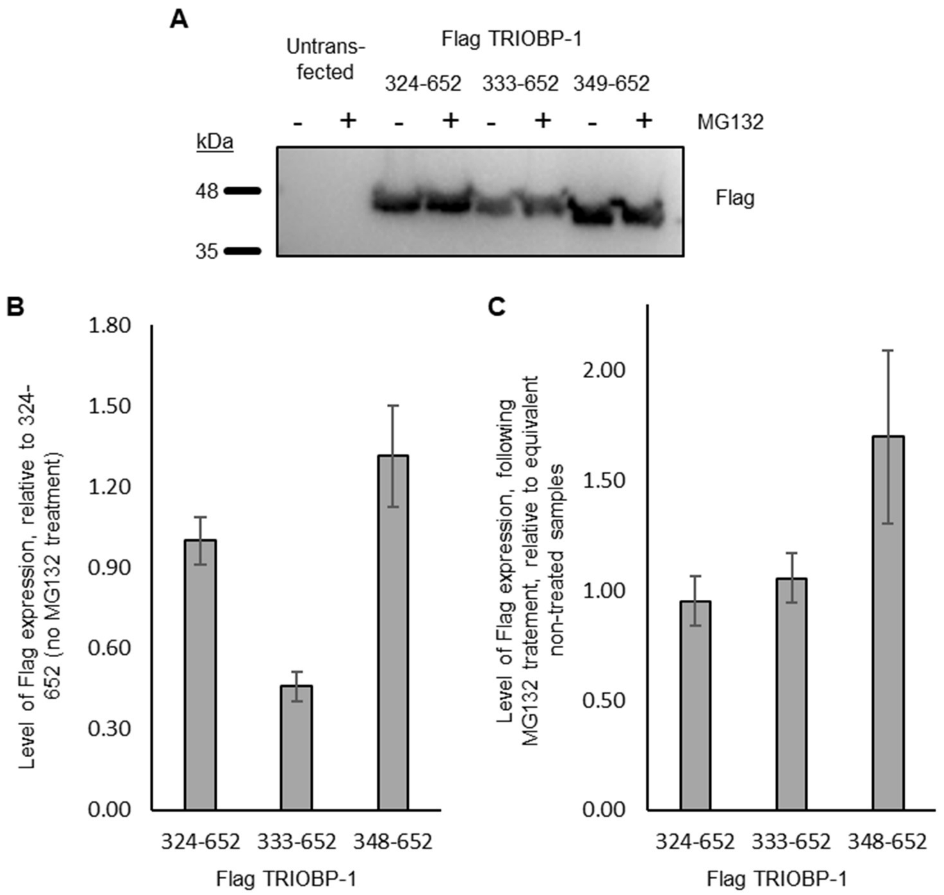


**Supplemental figure S5**: Low expression of TRIOBP-1 333-652, which cannot be rescued by inhibiting the proteasome. HEK293 cells were transfected with Flag-TRIOBP-1 amino acids 324-652, 333-652 and/or 348-652 (A) Example Western blot, typical of three replicates analysed here. (B) Graph displaying the expression level of each protein fragment, relative to 324-652. Expression of 333-653 is consistently lower than that of 324-652 or 348-652. (C) Graph showing the change in protein expression of each construct when MG132 is added, compared to a vehicle control. No significant change is seen, indicating that the reduced level of 333-652 in the cell does not arise because of proteasomal degradation.

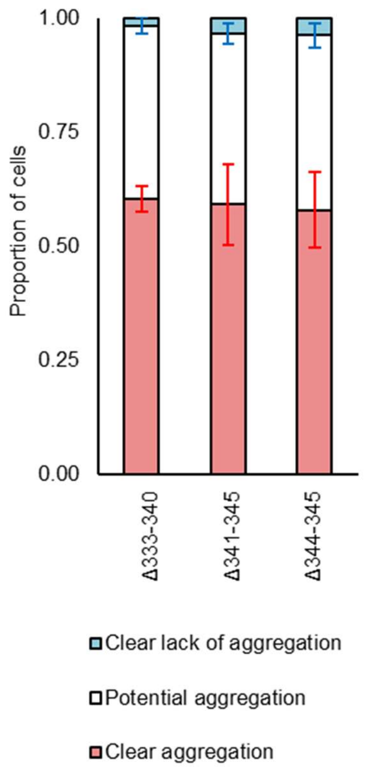


**Supplemental figure S6**: Quantitative analysis of TRIOBP-1 vectors with deletions of the central aggregation-critical site. SH-SY5Y cells were transfected with vectors encoding Flag-tagged TRIOBP-1 that was full length except for part of its central region. Images of typical cells are shown in figure 4B of the main text. Data from a blinded, quantified assay. Δ333-350: n = 58, Δ341-345: n = 57, Δ344-345: n = 53. Δ333-343 was also tested, but an extremely low level (n = 12) of surviving transfected cells meant that it could not be meaningfully analysed.
